# Challenges in the construction of knowledge bases for human microbiome-disease associations

**DOI:** 10.1186/s40168-019-0742-2

**Published:** 2019-09-05

**Authors:** Varsha Dave Badal, Dustin Wright, Yannis Katsis, Ho-Cheol Kim, Austin D. Swafford, Rob Knight, Chun-Nan Hsu

**Affiliations:** 1 Center for Microbiome Innovation, Jacobs School of Engineering, University of California, San Diego, 9500 Gilman Drive, La Jolla, CA 92093 USA; 2 Department of Computer Science and Engineering, University of California, San Diego, 9500 Gilman Drive, La Jolla, CA 92093 USA; 3 Scalable Knowledge Intelligence, IBM Research-Almaden, 650 Harry Road, San Jose, CA 95120 USA; 4 UCSD Health Department of Pediatrics, University of California, San Diego, 9500 Gilman Drive, La Jolla, CA 92093 USA; 5 Department of Bioengineering, University of California, San Diego, 9500 Gilman Drive, La Jolla, CA 92093 USA; 6 Department of Neurosciences and Center for Research in Biological Systems, University of California, San Diego, 9500 Gilman Drive, La Jolla, CA 92093 USA

**Keywords:** Natural language processing, Knowledge base, Microbes, Disease, Human, Model organisms, Microbiome, Microbiome dynamics

## Abstract

The last few years have seen tremendous growth in human microbiome research, with a particular focus on the links to both mental and physical health and disease. Medical and experimental settings provide initial sources of information about these links, but individual studies produce disconnected pieces of knowledge bounded in context by the perspective of expert researchers reading full-text publications. Building a knowledge base (KB) consolidating these disconnected pieces is an essential first step to democratize and accelerate the process of accessing the collective discoveries of human disease connections to the human microbiome. In this article, we survey the existing tools and development efforts that have been produced to capture portions of the information needed to construct a KB of all known human microbiome-disease associations and highlight the need for additional innovations in natural language processing (NLP), text mining, taxonomic representations, and field-wide vocabulary standardization in human microbiome research. Addressing these challenges will enable the construction of KBs that help identify new insights amenable to experimental validation and potentially clinical decision support.

**Electronic supplementary material:**

The online version of this article (10.1186/s40168-019-0742-2) contains supplementary material, which is available to authorized users.

## Introduction

**Fig. 1:**
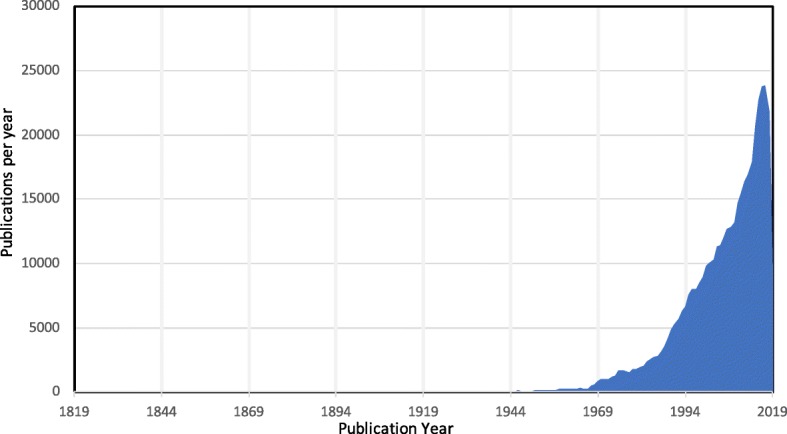
The rate of publications linking bacteria to human disease in PubMed. The chart displays the yearly count of PubMed abstracts matching human disease with microbes using the query (human AND disease) AND (microbiome OR microbiology OR microbes OR bacteria OR microbiota OR fungi OR virus). While the rise in publications began several decades ago, the last decade has featured a rapid increase in the number of publications spurred on by reductions in sequencing technologies and increased interest in the microbiome

The rapid decline in the cost of DNA sequencing, coupled with improved computational tools for interpreting DNA sequence data, has enabled microbes, humans, and other hosts to be genetically characterized at an unprecedented scale. High-throughput 16S rRNA gene sequencing and more recently shotgun metagenomic sequencing technologies now provide a means of efficiently identifying, classifying, and correlating microbiota with their environment [1–3]. Many new insights have been revealed by these methods, demonstrating that bacteria and other microbes such as archaea, yeasts, protists, and viruses play an important role in shaping human health and diseases [4–6]. These associations go beyond links between the pathogens that individually cause infectious diseases and include complex effects of the whole human microbiome on a range of different phenotypes. Accordingly, many laboratories have sought to determine the state of the “normal” human microbiome, especially in the gut, and characterize uncommon states as *dysbiosis*, presumed to be associated with both innate and lifestyle diseases [1, 7–9]. This has led to an exponential increase in the number of medical and experimental findings reported in publications linking microbes, including bacteria, to human diseases (Fig. 1). Understanding and drawing insight from this literature to establish human microbiome-disease associations for hypothesis confirmation and generation will be critical for scientific discovery going forward, but manually absorbing, interpreting, and curating the rapidly growing volumes of texts is beyond the scope of any individual.

The issue can be addressed with computer algorithms, such as *natural language processing* (NLP) and subsequent *text mining* techniques, to process existing repositories of text including abstracts from PubMed (https://www.ncbi.nlm.nih.gov/pubmed), full-text articles in PubMed Central (https://www.ncbi.nlm.nih.gov/pmc/), and topic-specific data repositories to extract and organize human microbiome-disease associations into a digestible form in *knowledge bases*.

A *knowledge base* (KB) is a large-scale structured repository of *entities* and *relationships* between them. One of the most successful biomedical KBs is the GWAS Catalog [10], containing entities of human genotypes and phenotypes and their relationships—whether a statistical association was observed between them in a published genome-wide association study (GWAS). The GWAS Catalog has become an essential resource for genetic researchers and clinicians to prioritize candidate loci and assess disease risk. The knowledge in that KB would be inaccessible otherwise without its users spending substantial efforts undertaking systemic reviews of the literature.

In a KB of human microbiome-disease associations, entities may include at least microbial organisms and disease/health conditions, while relationships between them may include promotion, inhibition, causation, correlation, and other types of associations. The huge volume of research literature is one of the largest information sources containing these entities and relationships, which are frequently, but not exclusively, built on experimental data deposited in repositories such as the European Bioinformatics Institute’s European Nucleotide Archive, the Joint Genome Institute’s Genomes Online Database, and the National Center for Biotechnology Information’s (NCBI) Sequence Read Archive. While these resources theoretically enable researchers to gain access to new discoveries through meta-analyses of 16S and whole-metagenomic sequencing data that combine information from many projects, in practice, this is infeasible as metagenomes are routinely provided without sufficient sample, preparation, and processing metadata to determine the conclusions reached by the researchers who designed the experiments, and for large data sets can further be computationally infeasible. While new platforms such as Qiita [11] will help to make this task more achievable in the future, extracting results directly from publications into a KB derived from publications bypasses this effort and provides a way to obtain information when a pure computational task is infeasible. Furthermore, this process leverages the insights provided by the researchers who designed the experiments and generated the raw data and includes almost all known high-quality, peer-reviewed study results that are available rather than limiting insight to studies where public sequencing data is available.

A major challenge to constructing a KB from the research literature is that these research articles and reports are written for human comprehension. Large-scale extraction of the information will require computerized NLP and text mining techniques to automate the process, thereby allowing the construction of a knowledge base to be sufficiently large and useful. NLP and text mining have largely matured in the general domains with [12–14] as prominent examples. These advances may potentially be translated into the use for human microbiome research and represent a promising avenue for the collection, curation, and normalization of human microbiome knowledge from the relevant literature.

Ideally, such KBs should contain all known human microbiome-disease associations from the research literature. However, many challenges must be addressed. Some of these challenges are generally applicable to any KB construction task while others are unique to the construction of human microbiome-disease association KBs. One of the most difficult challenges is how to identify and normalize various entities of interest (i.e., bacteria and disease) which depend on standardized nomenclature and ontologies. Otherwise, the computer may not know that “*Porphyromonas gingivalis*” are bacteria and may mistake “*P. gingivalis*,” “*Porphyromonas gingivalis*,” and “*Bacteroides gingivalis*” for different bacteria. Since the proof of germ theory ~ 150 years ago, conventions in naming, cataloging, and organizing diseases, microbes, and their properties have evolved considerably. Over the last two decades, new expected and unexpected discoveries [15] in human microbiome-disease associations have further blurred the line between the microbial host worlds and concepts of disease. For example, the statement “*Helicobacter pylori*-induced atrophic gastritis predisposes to gastric adenocarcinoma …” [16] illustrates a condition stated as a composite of bacteria and a disease and calls for advanced NLP techniques to correctly capture.

**Fig. 2:**
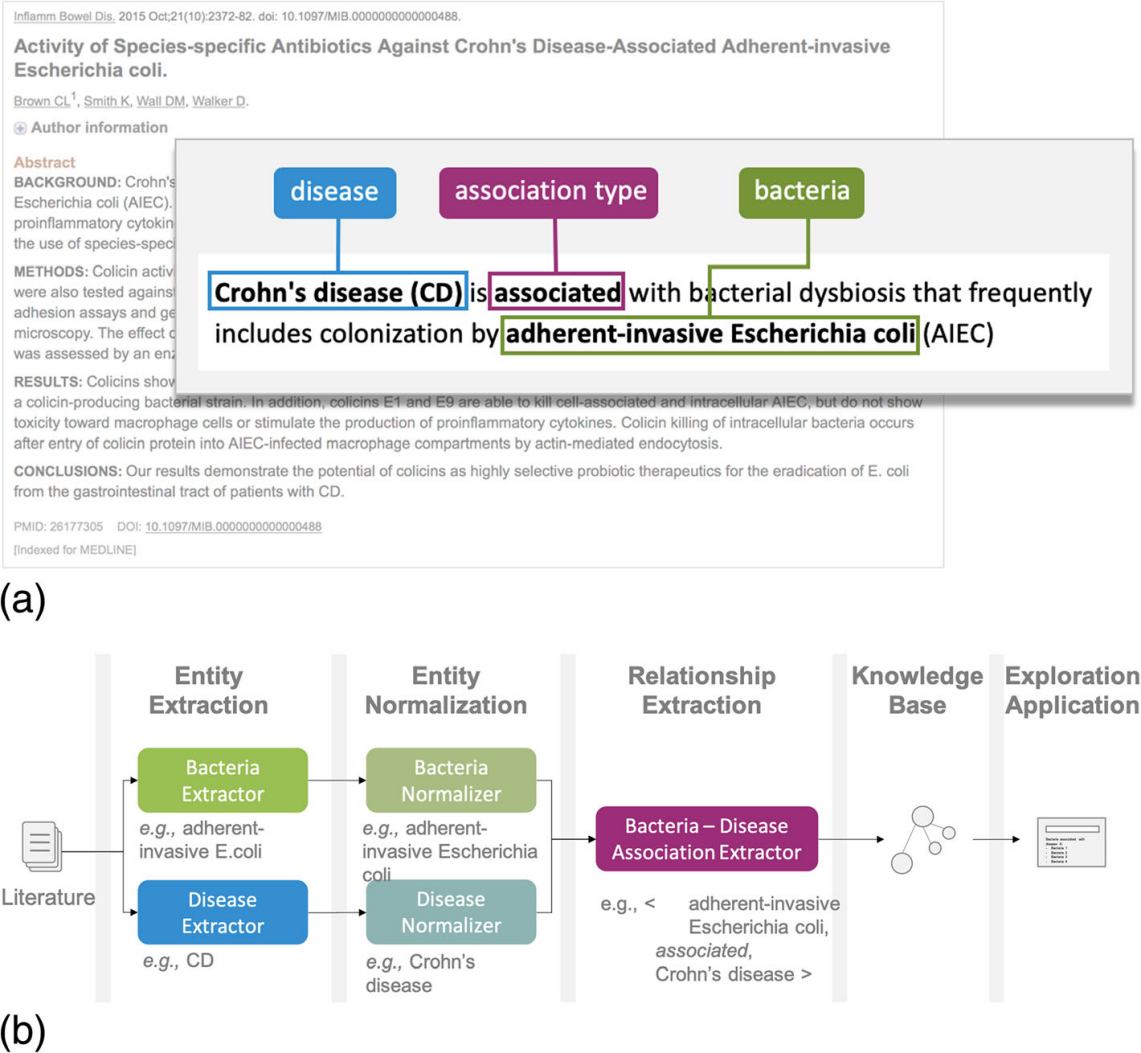
**a** An example free-text snippet in a publication where an association between a bacterium and a disease is stated and can be systematically extracted by NLP and text mining techniques to construct a knowledge base. **b** An overview of the essential steps of text mining the literature for the construction of a knowledge base of human microbiome-disease associations

Extracting association relationships between the human microbiome and diseases poses similar challenges and adds unique complications to challenge state-of-the-art NLP. For example, in the statement “Crohn’s disease (CD) is associated with bacterial dysbiosis that frequently includes colonization by adherent-invasive *Escherichia coli* (AIEC)” [17], one can ascertain that the microbe entity “adherent-invasive *Escherichia coli*” has a “positive association” relation with the disease entity “Crohn’s disease” (Fig. 2a). There are numerous potential association types that can be captured between the human microbiome and diseases. It is important to faithfully represent these associations with a well-defined and comprehensive classification for KBs to be useful.

This article reviews current efforts of constructing KBs of human microbiome-disease associations from the human microbiome literature, focusing on the areas for improvement in quality and validation and the associated challenges. Yandell and Majoros [18] provided an overview of the biomedical NLP techniques enabling a similar effort in genomics, where the key drivers are relations between genes, their sequences, and multitude of text fragments available across repositories of biomedical literature. The techniques reviewed may potentially be reused and extended here. We further suggest potential solutions to overcome these challenges including both technical approaches and community efforts to address the pressing needs of assembling and organizing human microbiome-disease association knowledge.

## Existing knowledge bases of human microbiome-disease associations

### Essentials of knowledge base construction

KBs of human microbiome-disease associations must at least contain two essential entity types: microbes (most frequently bacteria) and diseases. Microbes are organized under a hierarchical taxonomy (kingdom, phylum, class, etc.) of which usually lower levels (i.e., genus, species, and subspecies/strains) are predominantly referred to in the free text. The steps to obtain these relationships usually include (Fig, 2b) the following:

- Entity extraction, in which mentions of microbes and diseases are identified
- Entity normalization, in which extracted entity mentions are mapped to canonical identifiers (e.g., “CD” is mapped to “Crohn’s disease”)
- Relation extraction, in which the context of pairs of mentions is used to determine if a distinct relationship exists between the entities

Common approaches employ either pipeline (sequential) or joint (parallel) modeling of these three tasks. In addition, methods of verifying the validity and utility of the extracted results populated into the KB are necessary for the KB to be trustworthy and useful. More rigorous verification should involve integration of primary experimental data, e.g., metagenomic or metabolomic profiling of microbial communities associated with host diseases.

### Review of existing knowledge bases of human microbiome-disease associations

Remarkably, only four KBs that capture human microbiome-disease associations have been published, all in the last 2 years, with each limited in utility and the scope of the corpora used in their creation.

In 2017, Ma et al. [19] created a KB to capture the relationships between microbes and entities including diseases, genes, drugs, chemical fragments, and symptoms from a limited set of 61 publications. Few specific methodological details were reported beyond brief mentions of text mining and manual curation. This KB captured 483 directional (increase/decrease) microbe-disease associations between 39 human diseases and 292 microbes. To give a level of confidence in the validity of the extracted relationships, the associations were assigned a weighted score based on the number of publications supporting an association. The score incorporates a Log(*N*/*n*_*j*_) term, where *N* is the total number of diseases (here 39) and *n*_*j*_ is the number of diseases that are associated with microbe *j*. While a good first step, the number of microbes are orders of magnitude below the number in use in the microbiome research literature (~ 19,717, as of 2017 [20]), limiting the utility of this KB for the community.

In 2018, Song et al. [21] captured the relationships between diseases (hepatitis, conjunctivitis etc.), biomarkers (Prolactin, apoa-I, etc.), microorganisms (*Vibrio*, *Salmonella*, etc.), and host organs (lung, liver, etc.) using a correlation analysis on PubMed articles related to a predefined set of 18 diseases and 21 biomarkers. Disease, microorganism, and biomarker terms were expressed as word embeddings [22] using canonical correlation analysis (CCA) [23], which is a statistical method for dimensionality reduction. To validate the extracted relations, they assessed the degree of co-occurrence using the square root of the product of the number of publications with each disease and the number of publications with each marker appearing in their titles using the top 20 Google Scholar results from searching extracted pairs. They reported that 85% of highly correlated pairs appeared in these top results from Google Scholar, but only 15% of weakly correlated pairs appeared. However, this study is limited in the scope of diseases and microbes considered, as well as in the methods used to validate their associations.

Also in 2018, Janssens et al. [24] published the database Disbiome which links diseases classified using the Medical Dictionary for Regulatory Activities (MedDRA) system with microbes normalized using NCBI and SILVA taxonomies [24]. Disbiome also provides both direction and context for the associations, including information on how the microbes were identified [24] and providing answers to a survey of 16 questions that attempt to capture the quality of the reported associations. While Disbiome represents the largest and most comprehensive effort to date, covering nearly 200 diseases and ~ 800 microbes, it was based on manually assembled full-text publications associated with just 500 abstracts. Such an effort is not sustainable or scalable given the rapid pace of publication in this field as highlighted above and in Fig. 1. However, their effort does provide a solid framework for what kinds of information and standards a high-quality KB should present to users.

Most recently, in January 2019, Noronha et al. published the Virtual Metabolic Human (VMH) database, an extensively curated interdisciplinary database with multiple linked resources such as human metabolism, gut microbiome, disease, nutrition, and ReconMaps [25]. This database hosts details of metabolic pathways in human and gut microbes to enable visualization, investigation, and nutrition design. Cross-reference to other resources outside of this database such as BIGG [26], Biocyc [27], KEGG [28, 29], UniProt [30], etc. makes VMH easy to navigate and query [25]. VMH uses ReconMaps [31] to account for reactions occurring in organelle and the human metabolic reactions occurring in the cytosol and the extracellular space. While this effort will no doubt be a great resource for the field, it prioritizes depth over breadth, focusing on just 255 Mendelian diseases linked to the metabolic genes and reactions in the same catalog and 667 species with manually curated genome-scale metabolic reconstructions. This limited scope does not adequately capture the large number of microbe-disease combinations reported in the literature.

Although the four KBs that we surveyed above address microbe-disease associations, they are quite different in their intents and purposes. Ma et al. [19] illustrate the types of consistencies that are exhibited by relationships within the database. Song et al. [21], not surprisingly, demonstrate that disease microbe co-mentions in scientific publications imply the actual correlation between the two. Janssens et al. [24] bring about formal rigor in the identification of disease and microbial entities using specific ontologies. Virtual Metabolic Human [25] is designed as an exploration tool.

We searched the most recent publications on biomedical KBs, but to the best of our efforts, no new KB specifically developed to provide associations of human microbiome and diseases was found. The closest related ones among them were Editome Disease Knowledgebase, a database for RNA editome and disease associations that may help in understanding RNA editing machinery and thereby molecular mechanisms affecting diseases [32], and gcMeta, a data repository for archiving and publishing human and environmental microbiome samples integrated with web-based data analysis and workflow tools [33]. No information of the associations between the microbiome samples and diseases is provided explicitly.

As these publications represent the known work on end-to-end KB construction for human microbiome-disease associations, there is much room for improvement. In particular, there is a pressing need for new innovations to enable the automatic construction of a KB of human microbiome-disease associations.

## Extraction and normalization of human microbiome disease entities

Entity extraction, normalization, and relation extraction have seen rapid maturation in the general text domain [12–14], but there are still major challenges which need to be addressed in the biomedical domain as a whole and human microbiome-disease association in particular. Extraction and normalization of microbe and disease names begin with the creation of standard taxonomies that capture the canonical entities which the community has agreed to use. Standards for naming entities and for modifying the taxonomy must be in place as well in order to maintain consistency over time [20]. In addition to a standard taxonomy, there is a need for large-scale labeled datasets in order to provide wide-scoped ground truth data for training and testing methods. Table 1 gives an overview of the existing datasets of species and disease mentions that could potentially be used in creating these taxonomies. The complexity of each of these tasks depends on the domain of discourse and the *type* of entities, hence requiring a separate discussion of each.

**Table 1:** Existing datasets for disease- and species-related entities. Note that there are only two datasets which contain both diseases and species (miRNA and variome). In addition, species-level datasets are not specific to the human microbiome, so there is a need to create datasets curated for human microbiota

Dataset	Entity type	No. of annotations	No. of unique annotations
CDR [34]	Disease	12,694	3459
Variome [35]	Disease	6025	629
miRNA [36]	Disease	2123	671
NCBI Disease [37]	Disease	6881	2129
Arizona Disease [38]	Disease	3206	1188
SCAI [39]	Disease	2226	1048
CellFinder [40]	Species	435	51
Variome [35]	Species	182	8
miRNA [36]	Species	726	47
S800 [41]	Species	3646	1564
LocText [42]	Species	276	39
Linneaus [43]	Species	4077	419
BioNLP-ST 16 [43]	Species	619	277

### Microbe extraction and normalization

Methods for performing the extraction and normalization on microbial entities are presently limited by several features shared with general NLP and text mining tasks as well as several specific to the field. General challenges in NLP include recognizing mentions of entities of interest and normalizing those entities to canonical names. In the task of extracting microbial names, there is a paucity of effective tools targeted at recognizing their pattern of appearance, usage, and inherent hierarchical structure. Development of these tools is hampered by a lack of well-curated and labeled training examples, and a lack of a stable, defined, and controlled list of microbial names and synonyms.

#### Limitations of tools for microbial entity extraction

Microbial extraction tools generally use shallow parsing with feature engineering (i.e., conditional random field (CRF) [44–47]), and only recently with deep learning [48, 49]. In fact, though these systems attempt to perform microbial, specifically bacterial, entity extraction, the tools are designed to solve the general task of biomedical named entity recognition (NER) as opposed to developing models specifically designed for microbial extraction. Siu et al. [47] present a fast method for biomedical NER using character trigram features to perform rapid lookup in the Unified Medical Language System (UMLS) [50]. Habibi et al. [48] propose to use a bidirectional Long short-term memory (LSTM) network- and CRF-based model [51] for the general task of biomedical NER and obtain good results across entity types (disease, species, chemical names, etc.). In addition, Li et al. [52] propose a deep learning model which learns bacterial name recognition jointly with bacteria-habitat relation extraction. While useful for general species recognition, these models focus on the entities not specific to the human microbiome.

#### Limitations of annotated corpora of microbial entities

Obtaining ground truth data for microbial names is challenging due to the requirement of human expertise. As such, there is a lack of annotation tools, as well as annotators, to mark all of the microbial names from domain to subspecies level. Consequently, existing datasets with microbial entity annotations are sparse, containing only several hundred to a few thousand annotations and even fewer unique annotations (Table 1). In addition, these datasets contain microbes at varying levels of taxonomic resolution (e.g., phyla, genus, species, strain) across a broad spectrum of life and are not human microbiome specific. For example, the BioNLP Shared Task 2016 dataset [53], which contains mentions of bacteria, geographical places, and habitat from PubMed abstracts [54], contains many species related to plants and archaea, not reflecting the domain of human microbiota. This motivates the development of human microbiome-related annotation tools along the same lines as PubTator [55] as well as methods for encouraging active community engagement in providing new ground truth annotations. Such tools should also enable users to normalize microbial names as there exists no single standard taxonomy for microbes.

#### Limitations of existing microbial catalogs and taxonomies

Once a microbe mention is identified and extracted from a text, the mention must be linked to a microbe entity defined by a standardized taxonomy for entity normalization. In the domain of microbial naming, the usage of multiple, sometimes competing, and incomplete catalogs and taxonomies limits the ease of automatic entity normalization, as well as manual collection, curation, and normalization of information from the microbiome literature. Prominent microbiome resources [56] such as Bergey’s Manual of Systematic Bacteriology [57], Open Tree of life Taxonomy (OTT) [58], SILVA [59], RDP [60], Greengenes [61], and NCBI [54] differ in structure, organization, maintenance, and scope. This is the result largely of the methods of construction, i.e., manual or automated curation, choice or presence of phylogenetic trees, variation in sequence composition, and intended use of each [62]. The subsequent reliance on these resources created compounding divergence of information classification in the KBs previously highlighted.

Disbiome [24] linked the microbes to NCBI and SILVA taxonomy. Virtual Metabolic Human [25] linked microbes to external links such as NCBI taxonomy, KBASE [63], Uniprot [30], The European Nucleotide Archive (ENA) [64], Ensembl Bacteria [65], IMG [66], MicrobeWiki [67], and Genomes Online Database [68]. Ma et al. [19] manually curated microorganisms at the genus level. To help overcome these issues, in 1997, Jean P. Euzéby created the List of Prokaryotic Names with Standing in the Nomenclature (LPSN) now maintained and updated by Parte [20]. LPSN lists the names of prokaryotes from the International Journal of Systematic Bacteriology/International Journal of Systematic and Evolutionary Microbiology (IJSB/IJSEM) and attempts to provide the most up-to-date set of prokaryotic names while tracking the changes to names over time. The reliance of the field on a small collection of individuals, largely volunteer, efforts to ensure that microbial names, synonyms, and evolutions in conventions are captured and cataloged further provides motivation for the automated construction of KBs in the field of human microbiome research.

### Disease extraction and normalization

The task of extracting and normalizing human disease names faces many similar challenges as microbial extraction and normalization. More tools exist for disease entity recognition and extraction, but obtaining granular information and normalization remain key challenges, exacerbated by a surprisingly small set of annotated corpora. Progress is further inhibited by the existence and common usage of multiple, domain-specific taxonomies and ontologies for organizing disease names.

#### Limitations of tools for disease entity extraction and normalization

There are several systems that use the datasets listed in Table 1 to identify and then normalize disease mentions in text while a small number of tools have been developed for joint disease extraction and normalization.

DNorm [69] uses machine learning in conjunction with the MEDIC vocabulary. Disease mentions are located using BANNER [45] which is an entity recognition system based on a CRF. Text mentions of diseases are then represented using term frequency-inverse document frequency (TF-IDF) [70, 71] vectors for normalization. The names are normalized using pairwise learning to rank which outputs a set of MEDIC [72] concepts for extracted entities. DNorm achieved precision, recall, and F1-score of 0.8, 0.76, and 0.78, respectively, on the NCBI disease corpus [37].

TaggerOne [46] was a follow-up to DNorm by the same group. It utilizes semi-Markov models for joint entity recognition and normalization and is trainable for arbitrary entity types. Its performance on diseases from the NCBI disease corpus for NER was 0.82 F1-score, and its normalization F1-score was 0.8. TaggerOne compares favorably with state of art and is considered a strong baseline for the disease recognition and normalization task.

Our group recently developed the tool NormCo [73], which applies deep learning to the tasks of human disease recognition and normalization. NormCo uses a simple phrase embedding model with entity coherence that is achieved using a bidirectional gated recurrent unit (GRU) network in order to predict a coherent set of diseases within a document. The model shows strong improvements over TaggerOne on the NCBI disease corpus and comparable performance on the CDR dataset.

Other groups have also been exploring deep learning for NER and a multi-task learning framework for joint disease NER and normalization. A bidirectional LSTM network with a CRF output layer was presented in [48] for the task of biomedical NER. They show a strong performance across several datasets, indicating the potential of deep learning for the biomedical domain using the same neural network model as Habibi et al. [48] as presented in Zhao et al. [74], which shows improvements across recognition and normalization. All of the above methods rely on a source of well-curated and annotated training data with known disease names, synonyms, and hierarchies.

#### Limitations of existing human disease catalogs and taxonomies

Annotated, consistent, and comprehensive datasets for diseases, much like bacteria, are sparse, small-scale, and variable. For example, the largest dataset of diseases (CDR, see Table 1) contains only 3459 unique disease annotations, covering only 1082 unique disease concepts, whereas the Comparative Toxicogenomics Database (CTD) MEDIC dictionary contains 11,885 unique disease concepts (1082/11,885 = 9.1% coverage). In addition, a standard taxonomy does not exist for resolving disease names from text, though several have been attempted. One of the more popular taxonomies used across a variety of datasets is the CTD MEDIC dictionary [72] which is used across multiple datasets [34, 37]. CTD maps diseases to their canonical Medical Subject Headings (MeSH) or Online Mendelian Inheritance in Man (OMIM) IDs. Such a taxonomy is useful for research into knowledge base construction for human microbiome-disease associations, as shown in the Virtual Metabolic Human [25] which relied on 255 inborn errors of metabolism reported in OMIM for diseases. Other related disease taxonomies include UMLS [50], which integrates and distributes key terminology, classification, and coding standards, including SNOMED-CT [75], LOINC [76], RxNorm [77, 78], ICD-9 [79], ICD-10 [80], and many others.

These terminology systems were designed with different purposes and may not always serve the researchers’ purposes. For example, ICD was designed for statistical and reporting purposes, and as such, less common diseases may be lumped into a broad category, resulting in loss of information. In contrast, SNOMED-CT was designed for patient care documentation by clinicians and may better cover medical vocabularies used in verbal clinical communications. For example, ICD-10-CM diagnosis code E87.2 “Acidosis” cannot be classified further while SNOMED-CT contains at least three subclasses of acidosis: “metabolic acidosis,” “respiratory acidosis,” and “lactic acidosis.” SNOMED-CT clinical findings contain about 100K unique codes while ICD-9-CM contains only 14K and ICD-10-CM 68K. Among the 4 existing KBs, Janssens et al. [24] relied on an entirely different classification for their diseases using the Medical Dictionary for Regulatory Activities (MedDRA) classification system, highlighting the wide range of existing, inconsistent disease resources. Virtual Metabolic Human [25] integrates 21 external resources one of them being OMIM for diseases.

UMLS attempts to integrate these vocabularies by identifying subsets, mappings, and extensions and create lexical and mapping software tools. Therefore, in addition to identifying mentions of diseases through the comprehensive lists of aliases and synonyms in UMLS, it is also possible to take advantage of its 130 semantic entity types (e.g., “abdominal pain” is an entity of *symptoms and signs*, while “morphine” is a *clinical drug*) and 54 relations (e.g., “abdominal pain” is a symptom at the body part “abdomen”) for NLP to infer and extract disease mentions. Nevertheless, these taxonomies are general, containing entries for congenital diseases, disorders, and infectious diseases which may not be of interest to the community. Therefore, it may be necessary to curate a standard taxonomy of diseases specific to human microbiome-disease research that takes into account the interactions between microbes and their hosts.

## Extraction and representation of microbe-disease associations (relations)

Beyond entity extraction and normalization, it is necessary to build systems which can identify, ideally directional relations between entities of interest (i.e., microbes and diseases). Association (relation) extraction is usually a two-step process, requiring entity extraction at first. There are five major techniques that are usually employed for association extraction: (1) hand-crafted rules or slot grammars, (2) bootstrapping methods or semi-supervised learning, (3) supervised learning, (4) distant supervised learning, and (5) unsupervised learning. The first two techniques vary on how explicit the knowledge is coded, and the last three vary on how much effort is required for training. The training features themselves can be as simple as the presence of keywords, distance between entities, or something more complex such as a dependency parse tree.

The existing relation extraction approaches usually assume that a finite fixed set of relation types is given for NLP to assign the extracted relation to and that these relations are binary, that is, they are one to one. However, currently, no such set of association types for human microbiome and diseases is even in existence. As a result, there is a need to categorize and define the association types. We discuss the challenges involved in this association representation task from both technical and biological perspectives.

### Extraction of microbe-disease associations

#### Limitations of tools for extracting microbe-disease associations from the text

A simple approach to extract commonly occurring binary relationships is by using rules such as co-occurrence [81]. Slot grammars, in which entities are assigned to slots using template-based rules [82], allow for extracting relations of high order and complexity. Parse trees are widely used tools in the matching process to decompose a sentence into syntactic units, which can be used to deduce an implicit association implied from a sentence [82]. An advantage of grammar and parse tree-based approaches is that they do not depend on a large annotated corpus and may achieve a good precision, that is, the extracted associations are mostly correct. Their weakness is that these grammar rules and templates can hardly cover a wide variety of expressions that authors may use to state an association, resulting in a low recall, where too many stated associations in an input text are missed.

Recently, deep learning approaches have been proposed, for example by Verga et al. [83], who used a self-attentive transformer network to jointly learn entity recognition and relation extraction. Li et al. [52] described a neural network-based system to jointly perform entity recognition and relation extraction simultaneously to extract bacteria-drug associations. Though these deep learning approaches may generalize better to cover a wide range of expressions than rule-based approaches, their extraction performance results are still below 0.8 in terms of precision, recall, and *F*-score and may still be insufficient for high-quality KB construction. In general, relation extraction is still a challenging open research problem in all domains of NLP.

Also, existing works on relation extraction usually expect that the mention of a microbe in the proximity of a mention of a disease is strongly suggestive of an association. However, challenges may stem from anaphora and resolution of disease mentions. For example, authors may state that a certain bacterium exacerbates a symptom for patients of a certain disease instead of stating directly that the bacteria exacerbates that disease. This issue is related to the challenge of how to define and categorize a set of association relationships for KBs to fully capture the nuance of the connections as understood by human readers.

#### Limitations of knowledge graph completion

As the rapidly expanding body of knowledge about the associations between the human microbiome and disease shows no sign of decreasing with time, once this information has been parsed from purely human-interpretable into a KB, it should be possible to infer new associations from known associations. This computational task is known as *knowledge graph completion* [84] and has produced some interesting results in domains including protein-protein interactions [85–87]. The idea is that when a sufficiently large number of entities and the relationships between them are accumulated, we can consider them as a *knowledge graph* with entities as the nodes and relationships as the links in the graph. From the graph, it may be possible to induce signature patterns that can be used to accurately predict the presence of a relationship between two entities. Once the signature patterns are induced, we can then interpolate new relationships that are unknown between entities in the graph. Knowledge graph completion may complement NLP and text mining to increase knowledge base coverage and reveal latent relationships which may be difficult to extract from text.

Binary Matrix Completion MDA (BMCMDA) developed by Shi et al. [88] is a method that infers new microbe-disease associations from HMDAD developed by Ma et al. [19]. Similar work includes KATZHMDA [89], PRWHMDA [90], BiRWHMDA [91], and PBHMDA [92]. However, they share a common weakness that they have been evaluated on limited training data from HMDAD which provides a limited dataset that is far from sufficient as the ground truth. A larger KB with a sufficient number of reliable ground truth associations is necessary for a rigorous comparison of these knowledge graph completion approaches.

### Classification and representation of microbe-disease associations

Similar to the need of standardized catalogs and taxonomies for entity extraction and normalization, associations in a KB also need to be standardized. This section discusses the challenges of creating a standard to classify and represent microbe-disease associations.

### Challenges of creating a community-agreed classification of microbe-disease associations

There are a wide variety of associations between a microbe and a disease which have been observed and reported. A challenge is to classify these association relations into a set of useful and well-defined categories of association types. Intuitively, one may divide these associations into causation vs. correlation/inverse causation (e.g., reduced immunity resulting in bacterial growth) or negation of a previously reported relation. Potentially, we may apply either a knowledge-based approach (e.g., [93]) or a data-driven approach (e.g., [94]) to define and classify microbe-disease associations. As is the norm, Janssens et al. [24], Song et al. [21], and Ma et al. [19] model microbe-disease associations as qualitative directional (reduced vs. elevated) relationships only. This limits their usability in situations where disease progression may be associated with microbial blooms and possible cyclical replacements of communities of organisms. In a knowledge-based approach, we consider the mechanisms of how microbes interact with their hosts to create phenotypes and derive association types based on the underlying mechanisms. In a data-driven approach, we may extract a large collection of snippets from the literature where microbes and diseases are co-mentioned and cluster these snippets into semantically similar types to create a classification of associations. A hybrid approach can also be applied, but the resulting classification should be presented to the research community to request for feedback to ensure the quality and coverage.

### Challenges of representing stability and validity of associations

A major shortcoming of the existing approaches to entity and relation extraction for KB construction in NLP for general domains is that they usually assume that relations between entities are stable and permanent. However, human microbiome diversity and composition (i.e., species count and relative percentages) are not stable. In reality, human microbe populations can fluctuate tremendously, even over the course of a few hours, and may be influenced by factors including time since last meal, fasting, and passing of stool [95–97]. Variations in internal environment [4] due to diet [98–100], antibiotics, and host immunity all play an important role in shaping the gut microbiome [101, 102], and host genetics may influence both the microbiome and the progression of diseases such as inflammatory bowel disease [100]. Other factors, including microbial products such as lipopolysaccharides (LPS) and trimethylamine *N*-oxide (TMAO) may be important to capture when relating the presence or absence of microbes with specific diseases [100]. How to faithfully represent such conditional associations will need to be addressed to produce a high-quality KB with actionable information for informing human disease stratification.

A potential solution is to use dynamic models to model human microbiome dynamics to complement static association relations asserted in a human microbiome-disease KB. A dynamic model captures and expresses the interaction between microbes and human disease with mathematical models such as ordinary differential equations and Monte-Carlo methods as they can more precisely describe the quantitative and dynamic aspects of microbial associations [93]. Exactly how to construct a KB that represents dynamic associations is still an open research problem.

## Integration of knowledge base and experimental data

### Verifying the validity of KBs using experimental data

We can use experimental data to verify and validate the data extracted by NLP. Typical NLP and text mining tasks rely almost exclusively on manually annotated data as the ground truth to assess their accuracy and performance, but the tight link between microbiome data and human microbiome-disease interactions provides opportunities for exploiting experimental data as a source for these assessments as well as for generating new hypotheses. For example, we may use extraction tools and automated retrieval scripts to identify the connections of the data and analysis with EBI/ENA or Qiita [11] study identifiers and GitHub repositories or Qiime2 [94] objects with provenance. The structured text (i.e., tables and nested lists) of the per-sample metadata could then be used to verify the accuracy of disease mentions and normalization. Once the NLP-based approach is well-trained, the approach can be applied to the entire corpus of publications to extract knowledge that is not attainable in Qiita.

Likewise, reprocessing the experimental data with the provided scripts could confirm tagged microbe-disease association relationships as reported in the manuscript. Furthermore, by reprocessing the data using the latest best practices in microbiome analyses using tools like Qiita, it may be possible to identify the discrepancies between the reported and inferred associations based on the total human microbiome disease corpora. This would provide an objective, data-driven curation of associations while providing a metric of confidence in assessing the authors’ interpretations of the data presented in their articles.

We note that while the metadata available in Qiita covers the bacteria and related disease entities, the relationship associating them and more findings and insights that can be derived from publications will not be available from Qiita.

### Demonstrating the utility and expanding KBs with experimental data

A KB that enables an informative, insightful overview of all the published articles for a given field would not only allow users to stay current with the state-of-art but accelerates several universal tasks in research, including knowledge consolidation, new hypothesis generation, and identification of conflicting findings and biased data. We outline concrete examples of the use of a KB that repositories of experimental data do not provide alone and provide a worked example using the Disbiome knowledge base in this section.

#### Knowledge consolidation

A primary goal of a microbe-disease KB will be to consolidate lists of bacteria associated with the same disease in independent studies. As many bacterial names evolve and undergo reclassification, microbe-disease association KB can absorb such changes with minimal effort while allowing the continuity of associations to be maintained. A KB leveraging NLP to extract findings and insights from the publications will enable a rapid evaluation and prioritization of information that is impossible to achieve by simply scanning related publications or re-analyzing raw data scattered in multiple sources with inconsistent and variable metadata. The sheer amount and the growth rate of the publications demand an automated NLP approach.

#### Hypothesis generation

For KBs to have research or even diagnostic values, there must be clear paths to link primary analyses of human microbiome samples with information stored in the KB. As direct reference ranges for microbial populations can be difficult to establish and validate, inferring disease associations directly from experimental data are unlikely to succeed [103]. Instead, the KB may be used as a tool to suggest additional orthogonal testing of a microbiome sample, or the host donating the sample, to assist in the characterization of the diseases or conditions present. Over time, capturing primary experimental data through automated retrieval as discussed above and appropriate tagging of new primary experimental data used to query the KB may enable the appropriate embedding of single or multi-layer primary microbiome data as a component of the KB. For example, metagenomic and metabolomic analysis of a human microbiome sample could point to abnormal or modified host or microbial biological pathways. The inferred de novo associations can be cross-checked against patient symptoms or other data sources that capture specific pathway-host and pathway-microbe information. Indeed, there exist a large number of human-specific and microbe-specific databases that can be explored for integration into a unified and minimally cross-compatible KBs that capture information at the DNA, RNA, transcriptome, protein, metabolic, and/or structural levels [6, 104, 105].

#### Confirmation bias and contradiction

As a consequence of the knowledge consolidation inherent in a KB construction, the likelihood of bias can be examined by evaluating the proportion of associations that consist of self-citations or citations among networks of co-authors and collaborators.

In addition, the KB may readily expose biased information in the form of contradictory/conflicting findings between publications that deserve further investigation. For example, microbes shared across several etiologically different diseases are candidates for investigation of contamination or PCR bias, since it is unlikely for one organism to be a driver across so many different diseases. Sources of contradiction may include differential physical or statistical techniques for evaluating associations between microbes and diseases, potential unaccounted for variables, or spurious associations due to the use of contaminated laboratory reagents. This latter concern is best highlighted by the body of reports that PCR-based 16S rRNA gene sequencing and shotgun metagenomics methods can be impacted by contamination of microbial DNA during sample preparation due to molecular biology grade water, PCR reagents, or DNA extraction kits. Salter et al. reviewed various approaches to the problem [106]. Eisenhofer et al. [107] highlighted the impact of contamination, including contaminant DNA and cross-contamination, on microbiome research in low microbial biomass samples and recommended steps at different stages of an experiment known as the “RIDE” checklist. It is indeed feasible to match the microbe genera extracted by NLP from publications against the list of contaminated genera provided by Salter et al. [106] and Eisenhofer et al [107], as well as the more recent reviews to flag in the KB if a study may be driven by contaminants.

#### Worked example using the Disbiome knowledge base

In order to illustrate the above points, we selected two diseases with both overlapping and distinguishing clinical and etiological features: type 1 diabetes (T1D) and type 2 diabetes (T2D). Identifying common and divergent microbial profiles within each disease and between them could help researchers prioritize the avenues of research for microbe-host-disease interactions.

We began by selecting the list of organisms detected in “feces” from annotated experiments relating to each disease in Disbiome [24] and reduced this list to organisms annotated in > 1 experiment within the diseases or across the diseases (Table 2). This consolidation of knowledge enables us to quickly ascertain that of the 11 organism names annotated to be elevated in T1D, only 5 (the genera *Bacteroides*, *Blautia*, and *Veillonella* and the species *Bacteroides ovatus* and *Streptococcus mitis*) are annotated from multiple experiments. We can further see an evident contraction that 5 of the remaining 6 are also annotated as being reduced in T1D. Conversely, of the 12 organisms annotated as reduced in T1D, only 4 (the genera *Acidaminococcus*, *Dialister*, *Haemophilus*, and *Lachnospira*) have multiple supporting annotations while 5 have contradictory findings.

**Table 2:**
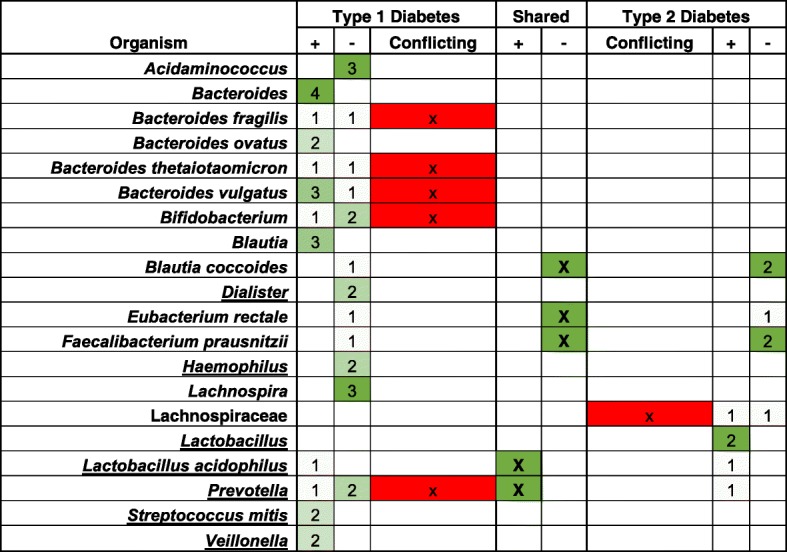
Findings from the Disbiome KB [24] for type 1 and type 2 diabetes with organisms annotated as elevated (+) or reduced (−) in “feces.” The counts represent the number of published experiments corroborating the findings as annotated in Disbiome

However, a quick comparison of the list in Eisenhofer et al. [107] reveals that one of the contradictory organisms, *Prevotella*, is a common contaminant, and likewise, four additional elevated microbes and two additional reduced microbes are also contaminants. After excluding the contaminants, the remaining bacteria with contradictory associations within a disease are excellent candidates for meta-analysis, identifying the difference in initial conditions, experimental methods, and/or bioinformatics and statistical techniques. Tools such as Qiita [11] and redbiom [108] may soon enable these analyses, though often incomplete or insufficient sampling, processing, and missing analysis metadata still make resolution of the contradiction challenging.

From the reduced list of microbes with non-contradictory evidence for their direction of association, a variety of ecological and etiological hypotheses can then be surmised by looking for common metabolic pathways, reports of competition between these groups, or impacts from medications or diet. Completing a similar comparison within T2D reveals only two organisms, *Blautia coccoides* and *Faecalibacterium prausnitzii*, that have multiple experimental annotations in the same direction (reduced). Intriguingly, these same organisms are reported to be associated with a reduced presence in T1D, albeit in a single experiment. This encourages the annotation of additional T1D and T2D reports to confirm this common association between the diseases and thereafter examine whether the reduction could be caused by the shared clinical features or perhaps causal, or evidence of causal impacts, in progression toward each disease.

While this example demonstrates the power of even a relatively small, manually curated, and annotated KB, implementation of the recommendations laid out in this review could enable even more potent knowledge consolidation, hypothesis generation, and bias detection. Indeed, within the expanded list of microbe names associated with each disease in Disbiome [24] used to create Table 2, there are several non-approved and reclassified bacterial names (Additional file 1: Table S1). Furthermore, by elaborating the full taxonomy of each name, we were able to uncover additional patterns of commonalities between T1D and T2D, as well as further contradictions (Additional file 1: Tables S2 and S3). Many of the contradictory and share bacteria were unsurprisingly reported to be contaminants as summarized in Eisenhofer et al.’s list [107]. All of these findings and inferences were performed manually, but demonstrate when an automated, robust, taxonomically aware, up-to-date KB can potentially bring about.

## Outlook and conclusions

We have reviewed an existing work on the automatic construction of knowledge bases of human microbiome-disease associations and identified essential technical challenges of this task. Based on our review and study, we summarize three key research thrusts that should be prioritized to accelerate the development of useful knowledge bases.

### Create common controlled vocabularies for bacteria, related entities, and associations

Common controlled vocabularies are essential to extract and normalize the various entity types so that the research community has a common yardstick to measure progress and share resources. Additionally, controlled vocabularies are important for the users of KBs. However, currently, commonly agreed vocabularies are missing, and many different ones have been used as reported in the literature. Bacterial names are typically available from LPSN [20] with detailed specification about their taxonomy, where each taxonomic category is a single word that allows for quick and easy lookup from a dictionary or hash table. In contrast, a CTD-like database [109] is available for diseases where a cursory examination is sufficient to realize disease names could be a single word or phrases with several synonyms and descriptions and proportionately many anaphoric references complicating the task. Also, it is crucial to develop a common classification of microbe-disease associations that covers known types of associations and to develop representations that capture dynamics of associations.

### Create and share large annotated text corpora to serve as the benchmark ground truth for the training and evaluation of the developed approaches

Similarly, large annotated text corpora are missing. This is a common issue shared by the whole biomedical NLP field because annotating biomedical text requires specialized expertise that is much more expensive than other NLP domains such as sentiment analysis of restaurant reviews. Though many semi-supervised and unsupervised machine learning approaches are developed to reduce the need of large volumes of annotated datasets for training machine learning algorithms, there is always a need to evaluate and compare the performance of NLP algorithms. Currently available benchmark datasets are too small (see Table 1) to reliably assess the performance when a system is applied to extract knowledge from, say, the entire set of PubMed abstracts. Resources need to be invested in the creation of such benchmarks as a community-based effort, combining the input of both expert and more-novice users to create a broadly relevant set of ground truth annotations with normalized and standardized taxonomies. This would further reduce the reliance on expert users for all annotation tasks as expert knowledge should not be required to identify the direction of an association between two domain-specific entities.

### Translate the recent advances in natural language processing from the general domain to various text mining tasks essential to automatic knowledge base construction in biomedicine

Primary techniques for entity extraction across various domains include dictionary lookup, rule-based, and supervised learning using CRF and deep learning, with the best-performing systems using hybrid approaches. State-of-the art systems achieve adequate accuracies, recall, and F1-scores for KB construction but require entity-specific implementation details. Advances in deep learning have enabled rapid improvements in entity recognition and normalization using a combination of BiLSTM and CRFs [48, 49, 110], and while these methods have begun to be translated into the biomedical domain, the rate of advancement in general domain NLP continues to accelerate. It will be important to investigate recent methods, especially pre-trained language models [14, 111, 112], which have shown dramatic improvements across language tasks, including entity recognition. Finally, relation extraction for microbes and diseases remains an open research problem. Most relation extraction systems rely on matching key terms and patterns in parse trees or phrases with templates. Few systems use word embedding-based deep learning approaches due to the lack of a large training corpus. Potential solutions for mitigating this lack of data include transfer learning [14], in which a model trained on a large general domain corpus is fine-tuned on a small corpus in the domain of interest, as well as multi-task learning [46, 74].

#### Potential issues and limitations

There are some potential issues and limitations that a KB derived from the literature may have that we need to be aware of when constructing a KB for human microbiome and disease associations. The KB will share the bias of the published literature. One of the well-known bias of the published literature is toward topics that are well funded; therefore, microbes and diseases that are intensively studied with rich funding will be covered more than those otherwise. Paywalls may be another issue for the construction of KBs but may be alleviated if publishers such as Elsevier and Springer Nature recognize that KBs add values to their contents and are willing to open text and data mining APIs to facilitate their subscribers to mine their paywalled contents automatically. Finally, eventually, the KB will need to cover non-English publications. For the moment, since NLP for languages other than English and machine translation are progressing rapidly, KB construction approaches developed for mining English publications can potentially be extended to other languages, though the amount of non-English publications at the moment may not justify the costs to include them. Moreover, non-English publications may already use Latin scientific names of bacteria for which a bacteria name entity extractor designed for English publications may still work with trivial changes.

In sum, the construction of a knowledge base of human microbiome-disease associations will be critical due to the rapidly expanding literature in the area, but the execution of this construction comes with several key technical challenges. In this article, we have identified the primary challenges, discussed the state of the art for each associated task, identified the limitations of current approaches and resources, and made our proposals for moving toward a fully realized KB of human microbiome-disease associations. It will be important going forward to identify specific entities of interest (both microbes and diseases), develop standard taxonomies of these entities, and curate relevant datasets in order to accelerate progress in this area. It is our hope that this critical survey will motivate the community to engage in more research on this important topic.

## Additional file

Additional file 1: Supplementary tables. (XLSX 39 kb)

## Data Availability

Not applicable.

## Abbreviations

AIEC: Adherent-invasive *Escherichia coli*
CCA: Canonical correlation analysis
CD: Crohn’s disease
CRF: Conditional random field
CTD: Comparative Toxicogenomics Database
ENA: European Nucleotide Archive
GRU: Gated recurrent unit
KB: Knowledge base
LPSN: List of Prokaryotic names with Standing in the Nomenclature
LSTM: Long short-term memory network
MESH: Medical subject headings
NER: Named entity recognition
NLP: Natural language processing
OMIM: Online Mendelian Inheritance in Man
OTT: Open tree of life taxonomy
T1D: Type 1 diabetes
T2D: Type 2 diabetes
TF-IDF: Term frequency-inverse document frequency
UMLS: Unified Medical Language System
VMH: Virtual Metabolic Human
